# VIVA (VIsualization of VAriants): A VCF File Visualization Tool

**DOI:** 10.1038/s41598-019-49114-z

**Published:** 2019-09-02

**Authors:** G. A. Tollefson, J. Schuster, F. Gelin, A. Agudelo, A. Ragavendran, I. Restrepo, P. Stey, J. Padbury, A. Uzun

**Affiliations:** 1 Department of Pediatrics, Women and Infants Hospital of RI, Providence, RI USA; 2 Department of Pediatrics, Brown University Warren Alpert Medical School, Providence, RI USA; 3 Data Science Practice, Brown University, Providence, RI USA; 4 Center for Computational Molecular Biology, Brown University, Providence, RI USA

**Keywords:** Software, Sequencing

## Abstract

High-throughput sequencing produces an extraordinary amount of genomic data that is organized into a number of high-dimension datasets. Accordingly, visualization of genomic data has become essential for quality control, exploration, and data interpretation. The Variant Call Format (VCF) is a text file format generated during the variant calling process that contains genomic information and locations of variants in a group of sequenced samples. The current workflow for visualization of genomic variant data from VCF files requires use of a combination of existing tools. Here, we describe VIVA (VIsualization of VAriants), a command line utility and Jupyter Notebook based tool for evaluating and sharing genomic data for variant analysis and quality control of sequencing experiments from VCF files. VIVA combines the functionality of existing tools into a single command to interactively evaluate and share genomic data, as well as create publication quality graphics.

## Introduction

Next generation sequencing produces an enormous amount of genomic data. This genomic data is stored in standardized data structures that have been designed to facilitate efficient analysis. The Variant Call Format (VCF) is a file format frequently used in sequence analysis^1^. VCF data visualization is an effective way to share experimental and genetic insights within teams and with external parties. Since VCF data is notoriously dense, effective communication of variant analysis results is needed. We believe that the variant analysis process can be made more transparent by making VCF interpretation more accessible to all clinicians and researchers interested in genetic data analysis.

**Figure 1:**
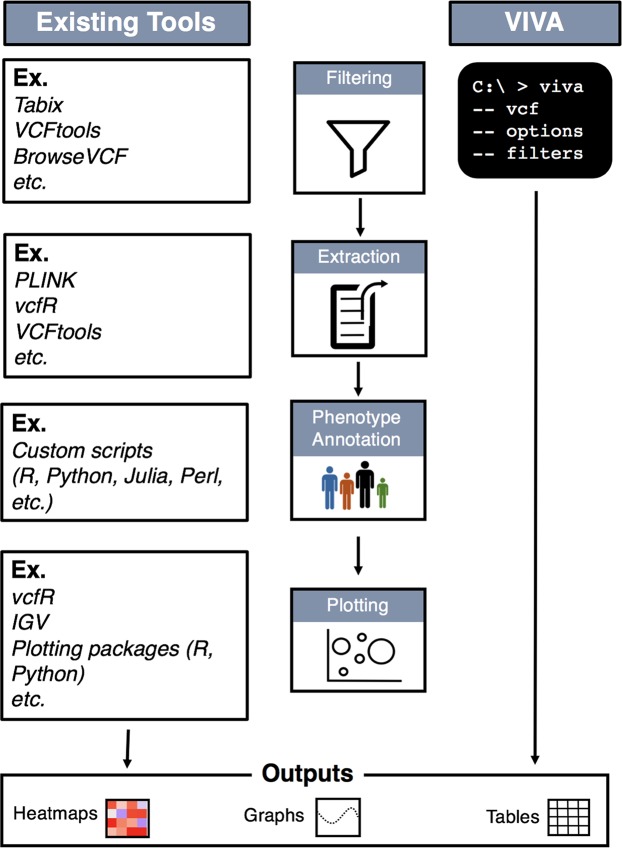
Comparative workflow. We present a general workflow for filtering, extracting, and visualizing variants from VCF files. This comparative workflow shows that while VIVA can perform filtering, extracting, annotating phenotype(s), and plotting functions in single command, other existing tools need additional and intermediate steps that require computational skills.

We introduce “VIVA”, a command line utility and Jupyter Notebook^2^ based tool for evaluating and sharing genomic data for variant analysis and quality control of sequencing experiments from VCF files. VIVA delivers flexibility, efficiency, and ease of use. VIVA’s functionality comprises three main parts: VCF file filtering, data parsing, and plotting. Currently, researchers must use a combination of tools to achieve this. Figure 1 shows an infographic comparing the current workflow with the workflow of VIVA to illustrate its contribution to this field.

Interpreting data from VCF files presents several challenges. The ability to process VCF files is limited by computational resources as the file size is often very large. To facilitate memory efficient data retrieval, existing VCF file parsing and visualization tools require users to preprocess their VCF files. This entails compressing and sorting VCF files by genomic position before either subsetting the file with an external program, such as VCFtools^1^, or indexing the files with Tabix^3^. Further, the VCF data structure is dense and difficult to interpret in its raw data format and requires data querying to draw insights.

We have prepared a detailed table of features comparing VIVA with some of the tools in the current workflow (VCFTools^1^, GEMINI^4^, BrowseVCF^5^, VCF.Filter^6^, VCF-Miner^7^, VCF-Server^8^, vcfR^9^, IGV^10^) (Table 1). We only considered tools that are open source and published in peer-reviewed journals. Our comparisons are based on four feature categories: technical details, filtering, visualization, and output options. In the technical details category, tools with the feature “one-step command” are defined as tools that can be run in a single step by the user. Tools that are run with a single one-step command load the input files, manipulate the file (for example filtering, extracting data, annotating with phenotype information) and produce output data (for example plots) without the need for intermediate user commands. The only tools that offer this feature are VIVA, VCFtools and IGV. All of the considered tools exist as standalone applications, except for VCF-Server. BrowseVCF and VCF-Miner have many of the same variant filtering features as VIVA; however, they have no options for visualization. Only VIVA and vcfR produce multi-sample heatmaps and read depth scatter plots. However, VIVA is the only tool which produces interactive HTML5 based visualizations, supports grouping of samples by like metadata traits, and displays multiple genomic regions and genotypic-phenotypic associations in a single plot.

**Table 1:** Comparison of features for VCF filtering and visualization tools.

Categories of Features	Features	VIVA	VCFtools	GEMINI	BrowseVCF	VCF. Filter	VCF-Miner	VCF-Server	vcfR	IGV
Technical Details	One-step command	✓	✓							✓
	Standalone software	✓	✓	✓	✓	✓	✓		✓	✓
	Environment (OS)	Windows, Mac, Linux	Windows, Mac, Linux	Windows, Mac, Linux	Windows, Mac, Linux	Windows, Mac, Linux	Windows	Windows, Mac, Linux	Windows, Mac, Linux	Windows, Mac, Linux
	Language	Julia	C++, Perl	Python	Python, JavaScript, CSS, HTML5	Java	Java	C, PERL‐CGI, JavaScript	R	Java
	Interface	Command Line, Jupyter Notebook	Command Line	Command Line, Web Browser	GUI, Command Line	GUI	GUI	GUI	R Console	GUI
	Docker container	✓	✓	✓			✓	✓	✓	✓
Filtering	Genomic ranges	✓	✓	✓	✓	✓	✓	✓		✓
	Variant position list	✓	✓	✓	✓	✓	✓			✓
	PASS filter	✓	✓	✓	✓	✓	✓	✓	✓	✓
	Sample selection	✓	✓	✓	✓	✓	✓	✓	✓	✓
	Variant annotations			✓	✓	✓	✓	✓		
	Dynamic filtering				✓	✓	✓	✓		✓
Visualization	Multi-sample heatmaps	✓							✓	
	Read depth scatter plots	✓							✓	
	Interactive HTML5 visualization	✓								
	Group samples by metadata traits	✓								
	Display genotypic-phenotypic associations	✓								
	Display multiple genomic regions	✓								✓
Output	Filtered results as tabular data	✓		✓	✓	✓	✓	✓	✓	
	Tabular output grouped by phenotype	✓					✓			
	Publication quality graphics	✓							✓	✓
	Export filtered VCF file		✓			✓	✓	✓	✓	

VIVA employs the Julia programming language, a high-level, high-performance, dynamic programming language for numerical computing^11^. VIVA is among the first user-level tools of its kind written in the Julia programming language. Additionally, it can be integrated into workflows with other tools hosted by BioJulia, the Julia language community for biologists and bioinformaticians.

## Methods

**Figure 2:**
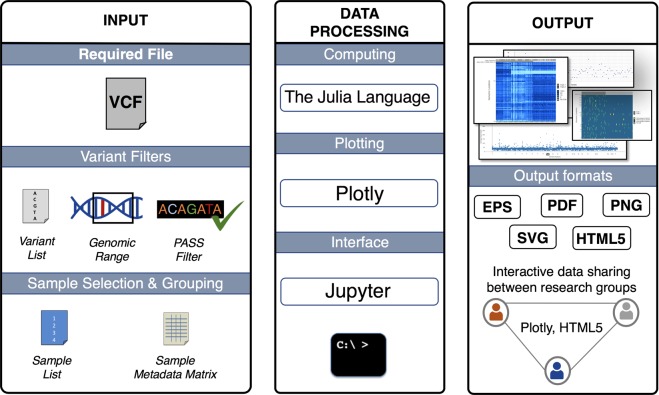
Workflow of VIVA. *INPUT*: VCF file is a required file. Users can use one or any combination of variant filters, sample selection, and grouping options. *DATA PROCESSING*: Data processing requires the Julia programing language and depends on several well-maintained Julia packages. Plotting uses the PlotlyJS.jl wrapper for Plotly. VIVA has two interface choices. Users may use the program through a Jupyter Notebook or from the command line. *OUTPUT*: VIVA’s four visualization options include heatmaps of genotype and read depth data as well as scatter plots of average sample read depth and average variant read depth data. These visualizations can be saved in HTML, PDF, SVG, or EPS formats. HTML format enables users to share and analyze the data interactively between research groups which supports collaborative work environments.

VIVA’s workflow involves three main steps which are illustrated in Fig. 2: (1) The user submits input files and chooses filtering options, if any are needed; (2) VIVA reads the VCF file and processes the data; (3) VIVA creates graphs and exports output files. These steps are all initialized in single command by the user.

### Data input

VCF is a text file format generated during the variant calling process that contains genomic information and locations of variants in a group of sequenced samples. The structure includes variant information such as genotype and read depth values for samples at each genomic position. The VCF file is the only required input file and it may be compressed or uncompressed. VIVA specifically supports VCF files for human genomes.

There are three optional text file inputs that support variant filtering and sample selection: (1) Variant List: A comma separated list of specific variant positions to include in visualization, including chromosome number and genomic position; (2) Sample Metadata Matrix: A .csv file with phenotypic or experimental metadata information. Any number of binary phenotypic traits or experimental conditions can be included in the matrix; (3) Sample List: A .csv file containing samples of interest to include in visualization. Examples of these input files are found in the repository hosted at https://github.com/compbiocore/VariantVisualization.jl.

### Variant record filtering

We recommend using one or a combination of VIVA’s variant filtering options when producing visualizations, as the number of data points included in visualization is limited by the user’s computational resources available for plotting and pixels needed for display. These filtering options include: (1) Pass Filter: Selects variant records that have passed filters selected during VCF generation; (2) Variant List Filter: Uses the Variant List input file described above to select variant records that match a list of genomic positions; (3) Genomic Range Filter: Selects variants that lie within a given genomic range (Ex: chr1:8900000–12000000). Users set filtering and visualization options in the command line interface or in the VIVA Jupyter Notebook utility’s settings.

### Sample ordering and selection

There are two options to manipulate the VCF data using sample IDs. Users can reorder the columns of the VCF file to explore trends across samples by supplying a matrix of sample metadata and sample IDs. Additionally, users can select specific samples to include in visualizations by supplying a list of sample IDs.

### Generating plots

We built our plotting functions using PlotlyJS.jl v0.12.3. We used this library to build heatmap functions for plotting read depth and genotype data, as well as scatter plot functions to create summary plots of average read depth values. Numerical arrays of genotype values are plotted by a heatmap function to produce a multi-sample categorical heatmap. These heatmaps display the genotype values: homozygous reference, heterozygous variant, homozygous variant, or no call for all selected samples and variants. These values are plotted with the Viridis color palette. Viridis is the default colormap of the Python plotting package, Matplotlib 2.0. Viridis is accessible to viewers with color blindness, visually appealing, and able to be converted to grayscale^12^. Read depth values are plotted in a continuous value heatmap using shades of blue that are reminiscent of ocean floor relief maps. For both heatmaps, y-axes are labeled with chromosome positions and x-axes are labeled with sample IDs. Users can choose to annotate their heatmaps with color bars showing binary sample metadata traits such as phenotypes or sequencing information.

File formats for saving heatmap and scatter plots include PDF, HTML, SVG, PNG, and EPS. Interactive plots can be saved in HTML to be used for real-time data exploration and are easily shared with other researchers who do not have VIVA installed. HTML plots are in HTML5 format and can be viewed in any browser. They support zooming, panning, and hover labels for real-time data exploration. Hover labels contain chromosome position, sample ID, and data values for each data point.

### Tool architecture

VIVA exists as both a command line tool and as a Jupyter Notebook hosted utility. Both of these tools are built with VariantVisualization.jl, our registered Julia programming language package for VCF file parsing, data manipulation, and plotting. VariantVisualization.jl depends upon a variety of other Julia packages. VariantVisualization.jl contains functions for data processing that are utilized in the following sequence: variant record selection, extraction of genotype or read depth values for selected variants into a numerical array, reordering the columns of the numerical array using sample metadata, selection of specific samples, and finally, plotting the resulting data.

The VIVA command line utility is implemented by calling the tool name, the name of the VCF file, and all usage options (‘viva -f file.vcf [options]’) in the Terminal or Windows PowerShell. VIVA options are evaluated and passed to VariantVisualization.jl functions by the ArgParse.jl v0.6.2 Julia package.

Our custom variant filtering functions utilize the GeneticVariation.jl v0.4.0 Julia package for reading data from VCF files. Our functions also utilize the VCF.Reader function to read VCF file variant records in the form of a reader object.

### Jupyter notebook

We used our VariantVisualization.jl Julia package to design a Jupyter Notebook with the full functionality of the command line tool. The VIVA Jupyter Notebook utility guides users who are unfamiliar with running bioinformatics tools on the command line through the use of VIVA. It includes a concise user manual in the first cell of the notebook. The next cells contain clearly labeled fields for entering the VCF file name and desired options. The user only needs to fill out the data input and option selection fields, then run the notebook to produce, save, and display interactive plots and publication quality graphics.

## Results

We compared VIVA to the current workflow needed to achieve comparable output (Fig. 1). As demonstrated in this figure in conjunction with Table 1, there is no single tool with which we can compare VIVA’s performance. One of our goals while building VIVA was to optimize the efficiency of reading and filtering large VCF files without the need for VCF file preprocessing. Thus, in the following section we compare the performance of VIVA to a VCF filtering tool (BrowseVCF). We also present use case examples of VIVA in which we visualize both differential burden of putative disease associated variants and evidence of batch effect.

### Benchmarks

#### Evaluation of performance

We evaluated VIVA’s performance on a MacBook Pro with 2.9 GHz Intel Core i5 CPU running macOS High Sierra with 8 GB 1867 MHz DDR3. Our test data set was a 13.58 GB VCF file from a whole exome sequencing study containing 6,699,236 variants for 100 samples. We ran VIVA and selected 8700 variants-of-interest from our test VCF file using default options to generate all plots. We saved four outputs, including a scatter plot of average sample read depth, a scatter plot of average variant read depth, and heatmaps of genotype and read depth values. All graphics were saved in HTML file format. We ran 5 replicates of this test and found it took an average of 4 minutes and 13 seconds with a range of 2 seconds.

#### Comparative performance analysis of VCF loading/filtering

We compared VIVA’s performance to that of BrowseVCF using 4 different simulation data sets. Two test VCF files were used, the first for Simulations 1 and 2 and the second for Simulations 3 and 4. In Simulation 1 and Simulation 3, the “PASS” filter was applied. In Simulations 2 and 4, the genomic range filter was used (chr3:1-4500000 and chr1:1-11134196 respectively). For all four simulations, VIVA’s runtime was shorter compared to that of BrowseVCF (Table 2).

**Table 2:** VIVA filtering runtime comparisons.

Simulations	VCF Filtering Simulations	VCF File Size	Number of Samples	Number of Variants	Number of Filtered Variants	VIVA Runtime (seconds)	BrowseVCF Runtime (seconds)
Sim 1	*PASS FILTER*	34.1 MB	24	37928	17901	25.97	109.29
Sim 2	*Genomic Range* (*chr3:1-4500000*)	34.1 MB	24	37928	60	27.30	96.00
Sim 3	*PASS FILTER*	261.5 MB	100	99850	99850	58.91	843.15
Sim 4	*Genomic Range* (*chr1:1-11134196*)	261.5 MB	100	99850	4498	31.50	672.28

### Application examples

**Figure 3:**
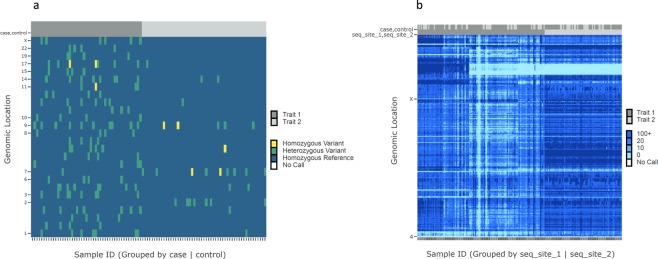
VIVA Use Cases. We present two use cases for VIVA. In both heatmaps, unique variant positions are stored in rows and individual samples are stored in columns. In the first use case (**a**) we visualize a differential burden of putative disease associated variants in a heatmap of genotype values for a set of 100 samples grouped by case and control metadata. In the second use case (**b**) we identify batch effect between a total of 191 samples sequenced at two separate facilities for a variant analysis study by visualizing read depth information and grouping samples by sequencing facility.

VIVA’s variety of visualization options creates many use cases for high-throughput sequencing experiment quality control and variant analysis. We present two such use cases in Fig. 3.

Grouping samples by phenotype or metadata is useful for comparative evaluation between samples. When sample grouping is implemented by the user, binary phenotype characteristics are visualized in a subplot of color bars at the top of heatmap visualizations. Users can add as many binary traits as they like to the visualization and can group samples by any one trait at a time. In Fig. 3a, genotype data is visualized in a heatmap for a select list of variants which had a statistically significant difference in distribution between the test groups. The samples are grouped by case and control. In this use case, the visualization highlights the differential burden of putative disease associated variants in the case group compared to the group of controls. In Fig. 3b, read depth values are visualized and samples are grouped by sequencing facility. In this use case, the visualization identified batch effect between groups of samples sequenced by the two sequencing facilities. There was a globally lower sequencing read depth achieved by one of the two sequencing cores, shown by areas of light blue indicating low read depth values.

## Discussion

We have taken several steps to make VCF file interpretation and visualization more accessible to researchers and clinicians. By consolidating the steps of current VCF file visualization workflows into a single tool, VIVA saves users’ time and resources. We have written comprehensive and clearly structured documentation with running examples to guide new users through their first runs. Since no coding is necessary to run VIVA, we have made VCF file visualization accessible to non-programmers.

VIVA was designed to optimize filtering speed using functions with a low-memory-footprint. VIVA reads through VCF files line by line and evaluates each variant record in the VCF file. The functions only load a variant record into memory if the record matches filter selection criteria. In this way, VIVA can read VCF files without loading the entire file into memory. Other tools require users to upload their VCF files onto a server for indexing and filtering. VIVA is both secure and fast because uploading large files onto an external server is not necessary. We showed in VIVA’s runtime comparisons (Table 2) that VIVA outperformed BrowseVCF in all simulations. We chose to compare VIVA against BrowseVCF because the authors of BrowseVCF showed faster performance than other filtering tools in their own comparisons^5^.

VIVA combines VCF file filtering and data extraction with flexible plotting functionality. Users can customize their plots by adding custom titles, selecting axes labelling options, and choosing from a variety of output file formats. VIVA supports interactive HTML output that can be used for real-time data exploration as well as scalable graphics formats like PDF and SVG for inclusion in publications and presentations.

VIVA’s array of visualizations can be used for quality control in sequencing experiments. Catching errors early on in the variant analysis workflow saves time and produces more accurate results. Plotting read depth values of sequenced samples across selected variants can help identify problematic samples and difficult to sequence genomic regions (both can be observed as very light blue streaks across the read depth heatmap in Fig. 3b). Batch effect can be identified with VIVA by grouping samples by user-supplied experimental metadata and plotting a heatmap of read depth values (as shown in Fig. 3b).

Sample grouping can also be used for exploring and presenting phenotype-genotype associations. By supplying phenotype metadata for samples, users can group samples by common phenotypic traits and plot variant genotype values. In this way, users can identify variants with higher distribution in samples with certain phenotypes. This could be useful for identifying variants that are associated with symptoms, with diseases, with drug effectiveness, and more. In this way, VIVA can be used for data exploration and presentation of findings in genetic disease research and precision medicine in the clinical genetics setting.

While our primary goal was to create a tool that is easily accessible to non-programmers, it is important to note that VariantVisualization.jl is an open-source package of clearly documented, modular functions. This allows other Julia developers to incorporate parts of VIVA’s functionality into their own projects. VIVA is one of the first user-level tools in the Julia programming language and sets a precedent for other command line tools to be built in the language. Functions within the VariantVisualization.jl package can be found at https://github.com/compbiocore/VariantVisualization.jl/tree/master/src.

Several improvements to VIVA are under development. We want VIVA to be accessible to users who prefer a graphical user interface (GUI). We chose to develop a Jupyter Notebook utility for VIVA to accommodate these users. Jupyter Notebook is an open source computational notebook that combines code, descriptive text, and interactive output and has become the computational notebook of choice with data scientists^2^. While a Jupyter Notebook makes running VIVA accessible to those who are unfamiliar with using the command line, in the future we intend to design an easy-to-use GUI for our application. Other VCF file filtering tools support dynamic filtering of VCF files. These tools are able to dynamically learn which fields exist in a submitted VCF file and allow users to filter by them. We intend to implement this functionality into a future version of VIVA. We also intend to expand our sample grouping feature to allow grouping of samples by more than one trait at a time. Finally, we are developing a variety of variant annotation features to support variant analysis and filtering by annotations.

## Conclusions

In conclusion, we have built a visualization tool for exploratory analysis and generation of publication quality graphics for variant analysis projects using variant call format (VCF) files. Researchers and clinicians can use VIVA to explore phenotype and genotype associations, batch effects on coverage, and differential incidence of variants between samples in their variant analysis experiments. VIVA provides a user friendly, one-step command and combines the functionality of existing tools to increase the accessibility of the current VCF file visualization workflow.

## Data Availability

VIVA is an open source tool built upon our registered Julia package, VariantVisualization.jl. It is freely available at https://github.com/compbiocore/VariantVisualization.jl. We have minted the project using Zenodo. The DOI is 10.5281/zenodo.3341840. Installation and comprehensive use instructions are detailed in the VIVA documentation which is available at https://compbiocore.github.io/VariantVisualization.jl/latest/. The tool undergoes routine cross-platform testing by the continuous integration services, Travis CI and AppVeyor. We have also implemented a Docker container to run VIVA without installing the Julia programming language and Julia package dependencies. Docker installation and use instructions are detailed in the manual at https://compbiocore.github.io/VariantVisualization.jl/latest/installation/#running-viva-with-docker-or-docker-compose.
